# Carbon-Based Nanocomposite Membranes for Membrane Distillation: Progress, Problems and Future Prospects

**DOI:** 10.3390/membranes14070160

**Published:** 2024-07-20

**Authors:** Chhabilal Regmi, Yuwaraj K. Kshetri, S. Ranil Wickramasinghe

**Affiliations:** 1 Ralph E. Martin Department of Chemical Engineering, University of Arkansas, Fayetteville, AR 72701, USA; 2 Research Center for Green Advanced Materials, Sun Moon University, Asan 31460, Republic of Korea; 3 Department of Energy and Chemical Engineering, Sun Moon University, Asan 31460, Republic of Korea

**Keywords:** membrane distillation, carbon nanoparticles, wastewater treatment, hybrid membrane, membrane fouling, wetting

## Abstract

The development of an ideal membrane for membrane distillation (MD) is of the utmost importance. Enhancing the efficiency of MD by adding nanoparticles to or onto a membrane’s surface has drawn considerable attention from the scientific community. It is crucial to thoroughly examine state-of-the-art nanomaterials-enabled MD membranes with desirable properties, as they greatly enhance the efficiency and reliability of the MD process. This, in turn, opens up opportunities for achieving a sustainable water–energy–environment nexus. By introducing carbon-based nanomaterials into the membrane’s structure, the membrane gains excellent separation abilities, resistance to various feed waters, and a longer lifespan. Additionally, the use of carbon-based nanomaterials in MD has led to improved membrane performance characteristics such as increased permeability and a reduced fouling propensity. These nanomaterials have also enabled novel membrane capabilities like in situ foulant degradation and localized heat generation. Therefore, this review offers an overview of how the utilization of different carbon-based nanomaterials in membrane synthesis impacts the membrane characteristics, particularly the liquid entry pressure (LEP), hydrophobicity, porosity, and membrane permeability, as well as reduced fouling, thereby advancing the MD technology for water treatment processes. Furthermore, this review also discusses the development, challenges, and research opportunities that arise from these findings.

## 1. Introduction

Water covers 70% of the Earth’s surface, and only 3% of it is fresh water; further, two-thirds of that is locked up in the ice caps or otherwise not suitable for human use. The rapid increase in the global population, the growth of cities, and the effects of climate change are all putting additional pressure on our water sources. Currently, around 1.1 billion people do not have access to clean water, and a staggering 2.7 billion people experience water shortages for at least part of the year. If we continue to use water at our current rate, this crisis will only escalate, leading to a situation where, by 2025, two-thirds of the world’s population will be dealing with severe water shortages [1,2]. Therefore, there is an urgent requisite for the development of water purification technology that consumes less energy and is environmentally friendly. Desalination has long been used to obtain freshwater from the sea or other saline water sources, allowing mankind to benefit from a potentially abundant supply of water. The MD process has become increasingly popular because of its unique advantages. Specially, MD is beneficial for small-scale decentralized water treatment systems in remote regions that struggle with infrastructure and lack centralized systems, particularly when combined with renewable energy sources [3]. The process is driven by heat and utilizes a microporous hydrophobic membrane to separate water vapor from saline water while efficiently removing non-volatile solutes. Based on the vapor–liquid equilibrium principle, the MD process exhibits a significant rejection rate. In theory, this method can eliminate 100% of non-volatile substances, ions, large molecules, particles, and microorganisms [4]. Additionally, its high tolerance to salinity allows it to effectively treat challenging hypersaline wastewater generated from oil and gas production, even surpassing the 80 g/L limit of reverse osmosis (RO) systems [5]. Nevertheless, the primary drawback of the MD technique lies in its significant energy consumption during the liquid–vapor phase transition. Furthermore, MD exhibits a lower permeated flux when compared to alternative separation processes such as RO, and its flux is greatly influenced by variations in the feed concentration and temperature, resulting in concentration and temperature polarization. These factors have a detrimental effect on the productivity and separation efficiency of MD. Similarly, the decline in separation performance over time due to pore wetting and the necessity for high thermal energy hinders its widespread application [6]. However, MD possesses the capability to effectively utilize low-grade waste heat as an energy source, function under low pressure conditions, and maintain a consistent performance regardless of variations in the feed salinity. This makes it a highly valuable alternative to traditional pressure-driven membrane processes. Additionally, MD can harness alternative energy sources like solar energy, thanks to its simple separation mechanism that relies solely on the transportation of water-vapor molecules through a hydrophobic membrane. The microporous membrane used in MD exhibits a high surface tension and hydrophobic properties, allowing only volatile vapors to pass through to the distillate section while preventing liquid from entering the pores. The transmembrane pressure in this process is generated by the saturation pressure difference resulting from the temperature gradient between the hot and cold sides [7,8,9].

## 2. MD Processes and Membrane Materials

The MD process differs from ultrafiltration and reverse osmosis as it involves not only mass transfer but also heat transfer. The efficiency of the mass transfer in MD is primarily influenced by the heat transfer. Therefore, understanding of the interplay between heat and mass transfer is crucial in unraveling the mechanism of the MD process. Within the process of MD, mass transfer occurs due to the variance in vapor pressures between the feed and permeating streams. The most prevalent setup for MD is DCMD, where the heated feed and cold permeate streams come into direct contact with the porous, hydrophobic membrane. The dissimilarity in temperature and composition of the solution in the layers adjacent to the membrane, between the feed and permeating streams, generates the driving force for vapor pressure [10]. The simultaneous occurrences of heat transfer and mass transfer lead to intricate heat transfer processes. Consequently, the mass transfer process has the potential to influence both the rates of the heat transfer and the coefficient of the heat transfer. The initial equation for the simultaneous transfer of heat and mass is obtained through the principle of energy conservation [11];

(1) $$q=JH\left\{T\right\}-kdTdx$$

where ***q*** is the total heat flux, ***J*** the mass flux, ***H{T}*** is the enthalpy at temperature ***T***, ***k*** is the thermal conductivity, and ***x*** is the distance in the direction of heat transfer. Due to heat transfer resistance, the temperature at the membrane surface differs from the temperature in the bulk. Thus, it is necessary to substitute the conduction term with the term for convective heat transfer in Equation (1) to demonstrate the heat transfer occurring in both the feed and permeating streams:

(2) $$q=JH\left\{T\right\}+h\left(T_{bulk}-T_{sur}\right)$$

where ***h*** is the convective heat transfer coefficient, ***T_bulk_*** is the bulk temperature, and ***T_sur_*** is the surface temperature.

Similarly, mass transfer in MD consists of three primary sequential stages: vapor generation at the feed–membrane interface, vapor transportation through the membrane pores, and vapor condensation at the permeate–membrane interface. The mass transfer in MD is governed by three fundamental mechanisms: Knudsen diffusion, Poiseuille flow (viscous flow), and molecular diffusion. Various forms of resistance to mass transfer can arise due to the transfer of momentum to the supported membrane (viscous resistance), collision between molecules (molecular resistance), or collision with the membrane itself (Knudsen resistance). Within this framework, the dusty gas model (DGM), which generally describes the gaseous molar fluxes through porous media, is generally employed to elucidate the mass transfer resistance within the MD system [12,13]:

(3) $$\frac{J_{i}^{D}}{D_{ie}^{k}}+\sum_{j=1\neq i}^{n}\frac{y_{j}J_{i}^{D}-y_{i}J_{j}^{D}}{D_{ije}^{0}}=-\frac{1}{RT}\nabla P_{i}$$

(4) $$J_{i}^{v}=-\frac{{\epsilon r}^{2}P_{i}}{8RT\tau \mu }\nabla P$$

(5) $$D_{ie}^{k}=\frac{2\epsilon r}{3\tau }\sqrt{\frac{8RT}{{\pi M}_{i}}}$$

(6) $$D_{ije}^{0}=\frac{\epsilon }{\tau }D_{ij}^{0}$$

where ***J^D^*** is the diffusive flux, ***J^v^*** is the viscous flux, ***D^k^*** is the Knudsen diffusion coefficient, ***D*^0^** is the ordinary diffusion coefficient, ***y*** is the molar fraction in the gaseous phase, ***p*** is the partial pressure, M_i_ is the molecular weight, µ is the gas viscosity, r is the pore radius, ϵ is the membrane porosity, τ is the membrane tortuosity, and ***e*** is the effective diffusion coefficient calculated by taking into consideration the structural parameters of the membrane as shown in Equations (5) and (6).

**Figure 1:**
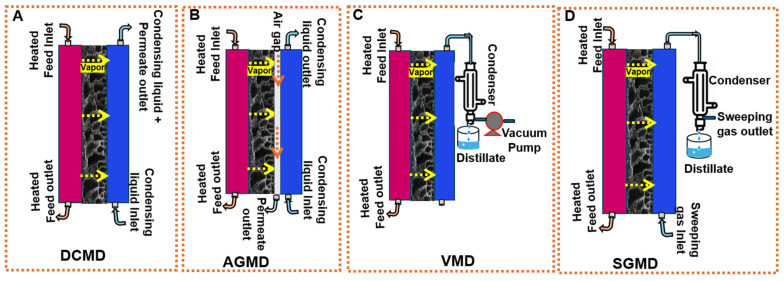
Schematic of four major MD configurations; (**A**) DCMD, (**B**) AGMD, (**C**) VMD, and (**D**) SGMD.

Based on the membrane module and the mechanism of heat and mass transfer, the MD processes are categorized into direct-contact MD (DCMD), vacuum MD (VMD), sweeping gas MD (SGMD), and air gap MD (AGMD). The schematic for different MD processes is shown in Figure 1. All configurations share the common feature of exposing one side of the membrane to the feed solution, leading to couple mass and heat transfer phenomena [14]. DCMD, with direct contact between the feed and permeation, is the simplest MD configuration and is widely used in desalination processes, serving as the basis for other MD processes. However, the excessive heat loss through conduction during its operation limits its applicability. This limitation can be overcome by using AGMD, although it introduces additional resistance to mass transfer as the permeation must pass through an air barrier, resulting in lower flux compared to DCMD. While the SGMD system reduces the heat loss and enhances the mass transfer coefficient, it also has its drawbacks. In this setup, a small volume of permeation diffuses into a large sweep gas volume, requiring a large condenser and increasing energy demand due to gas circulation. Similarly, the VMD system requires complex technical equipment to generate a vacuum and its operation has a higher demand for electrical energy, thus increasing costs [3,9].

Despite technological advancement, the popularity of MD is declining due to a scarcity of suitable membrane materials for distillation. The choice of membrane materials is pivotal for MD’s efficiency, significantly affecting the membrane’s performance and longevity. The key to enhancing MD is the development of superhydrophobic membranes. However, there are only a few intrinsically hydrophobic materials available. The most utilized polymeric membranes in MD include polypropylene (pp), polyvinylidene fluoride (PVDF), polyethylene (PE), and polytetrafluoroethylene (PTFE) [15]. Additionally, they often exhibit a reduced flux rate and are prone to issues such as wetting, fouling, and scaling, which can detrimentally impact the long-term efficacy of MD. To address the limitations of existing membranes not specifically designed for MD, new membranes with added functionalities, innovative structures, and unique morphologies have been developed. Some polymers, like polyamide (PA), polyacrylic nitrile (PAN), and polydopamine (PDA), that produce hydrophilic membranes have also been used for the MD process after undergoing hydrophobic surface modifications. Apart from the polymeric membranes, some hydrophilic ceramic membrane like alumina, silica, and zirconia after hydrophobic surface modifications are also used for the MD process. However, the intricate processes and sophisticated methods required in manufacturing these membranes can lead to high expenses and lengthy production times, diminishing the appeal of these ceramic membranes for widespread industrial use [16,17].

## 3. Necessity of Nanocomposite Membrane

Considerable progress has been made in developing high-efficiency membranes for MD. However, issues like reduced permeate flux, membrane fouling, and pore wetting remain prevalent in MD processes. These obstacles can be overcome by integrating advanced nanomaterials such as carbon-based nanomaterials, metalloids, metal-oxides, metal–organic frameworks (MOFs), and quantum dots into the membrane composition, thereby customizing the materials and the structure of the membrane [18]. Researchers have enhanced membrane matrixes by embedding nanoparticles (NPs), forming nanoporous structures that expand the surface area and add extra channels for water-vapor transportation. This leads to a greater vapor flux and improves the efficiency of separation. Incorporating nanoparticles or modifying the surface of membranes can reduce fouling and enhance the membrane performance, including an increased flux, better salt rejection, improved hydrophobicity, and superior physical and chemical properties for enhanced functionality [19]. Nanomaterials primarily regulate the wettability of the membranes by altering the surface morphology or imparting self-cleaning capabilities, reducing surface pollutants, and preventing membrane wetting in solutions containing contaminants [20]. Most modified MD configurations aim to either minimize the heat loss by reducing the temperature polarization or enhance the mass transfer and permeate flux. The permeation flux in MD is closely linked to structural characteristics, including porosity, thickness, pore size, and tortuosity [21]. The permeating flux tends to rise with an increase in the membrane pore size and porosity, which is attributed to the greater surface area available for transfer. Conversely, it typically decreases with a higher tortuosity and thickness due to an increased mass transfer resistance and reduced heat loss [9,22,23]. Recently, researchers have shown a growing interest in functionally graded nanostructured membranes. These membranes, which are enhanced by incorporating nanoparticles or by nano engineering of their surface structures and morphologies, improve upon the properties of the original membrane and stimulate the process performance. They are fabricated by modifying existing commercial membranes or by directly constructing functionalized membranes from their precursors. Studies have extensively covered these membranes in various forms, including flat sheets, hollow fibers, and nanofibers membranes [24]. Based on the location of the nano fillers in the membrane, nano-enabled membranes for MD can be categorized as follows [20];

- Conventional nanocomposites where the NPs and polymer matrix are homogeneously blended.
- Surface-modified nanocomposites, which involve the positioning of nanofillers on the surface of the membrane, thus altering the surface’s morphology and properties.
- Thin-film composites with nanocomposite support, where the thin separate hydrophilic layer of a polymeric dope solution is coated on the surface of the conventional nanocomposite to enhance the antifouling properties of the membrane.
- Thin-film nanocomposites, where the polymeric membrane’s surface is covered with a thin coating of a conventional nanocomposite layer which primarily enhances the antifouling properties.

For the successful integration of nanomaterials into polymer nanocomposites, it is crucial to ensure sufficient dispersion and proper interfacial adhesion with the polymer matrix during the modification process. Intrinsic properties like the particles’ size and morphology, Van der Waals forces, and the aspect ratio of the NPs are liable for their agglomeration. The characteristics of nanocomposites are influenced, in part, by how well the filler is dispersed and how it interacts with the matrix. Different physical dispersion methods like sonication, stirring, milling, or calendaring, as well as chemical dispersion like solvent dispersion and chemical functionalization, can be implemented. Successful homogeneous incorporation of the fillers in polymer matrices facilitates boosting up the microstructural as well as separation properties [25,26]. Choosing the right nanomaterials as fillers or additives and an easy preparation method are crucial for developing nanocomposite membranes for the MD process. The nanoparticles’ compatibility with the polymer matrix is also vital. If the materials are incompatible, it can reduce the membrane’s liquid entry pressure (LEP) and resistance to wetting. A pivotal step in manufacturing is selecting materials that have effective binding agents. NPs that have functional groups which can create strong connections with polymers are particularly important. This helps maintain an impeccable microscopic structure in the final product. Additionally, the nanofillers’ physical and chemical characteristics significantly influence the membrane’s efficacy [20].

In recent years, the focus of many researchers has shifted towards nanocomposite membranes, especially those incorporating carbon-based nanomaterials, for the development of advanced MD-based water treatment systems. It seems that these carbon-based nanoparticles play a dual role, as they not only help in forming a layered structure but also serve as binding sites for a coupling agent, resulting in the formation of a strong, uniform, and water-repellent film. The carbon atom has an electronic configuration of 1s^2^,2s^2^,2p^2^, allowing it to form several allotropes. Its narrow 2s and 2p orbitals can easily promote an electron, leading to hybridization into sp, sp^2^, and sp^3^ structures. These structures enable carbon to create diverse molecules. For instance, sp^3^ hybridization produces a tetrahedral structure found in diamond, which is hard, clear, thermally conductive, and electrically insulating. On the other hand, sp^2^ hybridization creates graphite’s layered structure, which is soft, opaque, and thermally and electrically conductive, with layers held together by weak Van der Waals force. A single layer of graphite is graphene and, when rolled in a cylinder, it forms carbon nanotubes (CNTs). The presence of both sp^2^ and sp^3^ bonds leads to a fullerene or buckyball formation, resembling a soccer ball. Carbon also exists in an amorphous form [6,27]. Graphyne is a novel 2D carbon allotrope composed of sp and sp^2^ hybridized atoms. It possesses outstanding mechanical and structural properties that could benefit the desalination process through its practical applications [28]. The various atomic configurations result in different allotropes with unique properties. Overall, these low-dimensional carbon-based nanomaterials exhibit distinctive properties like tunable chemical functionalities, a high specific surface area, high strength, tunable hydrophobicity, enhanced vapor transport, high thermal and electrical conductivity, considerable mechanical strength, and a large adsorption capacity [6,29]. Functionalizing these substances chemically is a widespread method to enhance their attributes, improve interaction with the matrix, and facilitate processing in solutions. Their application in MD has led to better membrane functionality, marked by a higher permeability and diminished fouling [6].

**Figure 2:**
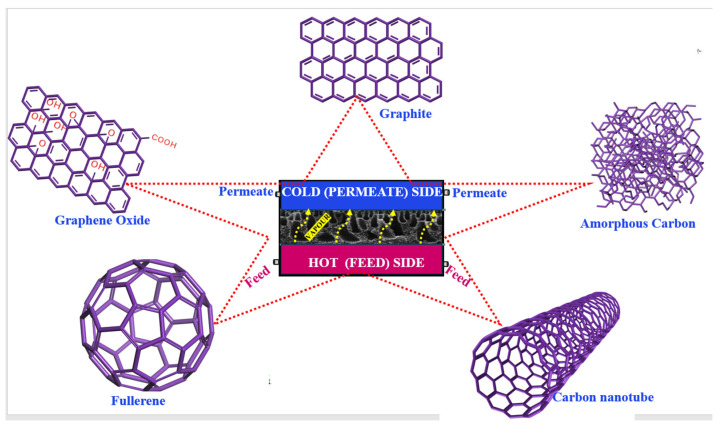
Different types of carbon nanomaterials in MD process.

Carbon nanomaterials such as activated carbon, carbon black (CB), graphite, CNTs, graphene, etc., are promising building blocks for membrane-based separation because they have unparalleled and exceptional mechanical, chemical, and thermal stability, as well as conductive and antibacterial properties [30]. Such materials enable a noble composite membrane with high productivity, long-term stability, and low energy efficiency that is especially applicable for the thermal-driven MD process [31]. The structures of some of the commonly used carbon nanoparticles in the MD process are given in Figure 2. The use of such low-dimensional carbon materials in MD has not only enhanced membrane functions, such as increasing permeability and reducing fouling, but also introduced new features like real-time fouling detection, targeted heat generation, and membrane cleaning. CB and activated carbon, two types of amorphous carbon with a large specific surface area, provide a high absorption capacity for various molecules. Moreover, these materials possess outstanding photothermal properties, allowing them to efficiently absorb sunlight and transform it into heat [32,33]. Unlike amorphous carbon, graphite, with a crystalline structure, possesses a low surface area but higher thermal and electrical conductivity [34]. Graphene-based nanomaterials, including graphene oxide (GO) and reduced graphene oxide (rGO), are paving the way for advancements in MD membranes due to their distinctive properties and straightforward synthesis methods. GO, characterized by its non-oxidized carbon atom networks and a plethora of oxygen-rich functional groups (such as hydroxyl, carboxyl, and epoxy) within its carbon framework, has demonstrated exceptional permeability and resistance to fouling. Moreover, integrating such hydrophobic nanomaterials into the membrane matrix has been shown to improve pore wetting and reduce heat loss across the membrane [35]. Many researchers have been inspired by the natural development of superhydrophobic surfaces to replicate the micro- and nano-scale structures found on these bio-surfaces [36]. Meanwhile, rGO has a lower oxygen content and exhibits distinct electronic properties while retaining similar mechanical and thermal characteristics. Despite its high conductivity, and although it has less wettability and dispersibility, its greater hydrophobicity compared to GO makes it a promising material for enhancing membrane properties in the MD system [37]. Likewise, CNTs exhibit distinctive characteristics suitable for membrane-based separation, such as a high aspect ratio, an atomically smooth surface, and a hydrophobic inner channel that facilitates a high slip length and low-friction water/vapor transport. Additionally, their chemically active tips allow for pore functionalization to tailored the membrane’s filtering properties [38,39,40]. Bucky paper (BP) is a thin sheet made of a self-sustaining film of randomly arranged CNTs, resembling black paper in appearance. It features a porous, entangled network structure as free-standing CNTs film. Typically, it is prepared through the vacuum filtration of the well-dispersed CNT solution. BP’s porous structure makes it suitable for a broad array of potential uses [41]. Membranes synthesized using bucky paper display advantageous characteristics for MD, such as significant hydrophobicity, extensive porosity, a large specific surface area, and comparatively low thermal conductivity. These characteristics suggest that BP membranes could outperform conventional PTFE/PVDF membranes, which are typically used as benchmarks in MD processes. Owing to their distinctive properties, various carbon materials have thus garnered considerable attention for the development of novel carbon composite membranes in the MD process [42].

The sustainability of low-pressure MD in water treatment is restricted by membrane fouling, causing a reduction in the membrane’s permeability due to the accumulation of aquatic components on the membrane surface or within the pores themselves. The development of low-pressure membranes that possess a strong resistance to fouling could prove to be highly beneficial, given that these membranes would necessitate less frequent chemical cleaning and demonstrate an extended operational lifespan [43]. Carbon nanomaterials can be utilized to modify the surfaces of existing membranes through coating techniques, layer-by-layer assembly, or chemical grafting methods. These nanomaterials can be immobilized on the membrane surface through Van der Waals interactions, charge interactions, or chemical bonding. The covalent cross-linking of carbon derivatives to the membrane surface is typically achieved by utilizing carboxylic and hydroxylic groups on the surfaces of carbon nanomaterials. This process ensures long-term stability and prevents nanomaterial leaching during operation [44]. Carbon nanomaterials possess a wide range of chemical properties and antibacterial characteristics which render them highly useful in addressing issues related to scaling, the membrane’s colloidal properties, and biofouling [44]. Incorporating such nanomaterials into a thin selective layer during the interfacial polymerization process enhances the membrane’s negative charge by introducing a significant number of carboxylic groups. Additionally, it results in the formation of supplementary transport pathways for water/vapor transport by disrupting the packing of polymer chains [45]. Similarly, the integration of carbon nanomaterials into polymeric membranes or the utilization of nanomaterial thin films as the primary filtration layer has been recognized as a viable approach, offering potential for the advancement of energy-efficient, compact desalination systems. The nanoporous configuration facilitates rapid water/vapor flow through precisely defined channels, with the membrane’s permeability being influenced by both the pore density and membrane thickness [46]. The functionalization of the membrane with these carbon nanomaterials also demonstrates outstanding antimicrobial properties. It can absorb phospholipids from the bacterial membranes onto its surface. This disruption of the bacterial membrane integrity leads to the discharge of essential intracellular components, ultimately leading to microbial inactivation. Likewise, the generation of oxidative stress by nano-sized carbon materials represents another significant antimicrobial mechanism [47]. Carbon-based membranes are also crucial in the development of anti-adhesion membranes. The addition of carbon-nano materials like GO into polymeric membranes promotes the formation and expansion of the polymer, leading to a more even membrane surface. Upon grafting GO on membrane surfaces, Hegab et al. observed that the GO molecules filled the surface valley, resulting in the flattening of the surface [48]. A smoother membrane surface, achieved through the incorporation of such carbon-based materials, decreases the surface roughness, lowers the surface charge, and minimizes biofouling [49].

## 4. Fabrication of Carbon-Based Nanocomposite Membrane

### 4.1. Free Standing Carbon Membrane

The synthesis of self-standing membranes for water purification applications has been gaining interest. Ultrathin, nanoporous 2D materials with a thickness of just one or a few atoms, combined with superior mechanical strength, are viewed as optimal components for creating thin membranes. These membranes offer minimal resistance to transport and maximum permeance, making them ideal for sea-water desalination. Carbon-based membranes that stand alone, without any supporting layers, possess distinctive structural and surface characteristics that hold considerable promise for desalination processes. The absence of a supporting layer in theses free-standing structures not only makes the thin membrane more adaptable for various water treatment applications but also minimizes internal concentration polarization (ICP), thereby enhancing their performance [50]. Self-supporting CNTs, known as BP, have attracted attention as a free-standing membrane for the MD process. The tangled network of CNT ropes and bundles is held together by non-covalent interactions. The remarkable capabilities of BP are largely due to the exceptional characteristics of individual CNTs, which include an extremely high Young’s modulus and superior thermal and electrical conductivity. Moreover, the paper-like form of BP provides great flexibility and an efficient heat transfer performance [51]. Dumée et al. developed a self-supporting CNT BP membrane by a vacuum filtration process. The hydrophobic membrane, with a contact angle of 113°, showed 99% salt rejection in a DCMD setup. They suggested that greater hydrophobicity led to better vapor transport. A decline in flux with time and membrane aging by delamination were some of the limiting factors [52]. Similarly, in free standing GO membranes developed by Nair et al., consisting of mixture of GO and rGO nanosheets, the nanochannel formed between the GO and rGO nanosheets served as a selective barrier, allowing the water vapor or gases and ions smaller than the channel diameter, while blocking other larger species [53]. Gong et al. developed a graphene-based free-standing foam that absorbs light and used it to create a new type of solar vapor-generating system. This system efficiently transports water, heats locally, and separates the membrane from the feed solution. When exposed to sunlight, the foam converts solar energy into heat, which is then transferred to a thin layer of water around the foam. This process results in localized heating and the effective production of clean vapor. This design’s uniqueness allowed for an impressive solarwater energy efficiency of 73.4%, strong resistance to fouling, and consistent performance over a period of 72 h [54]. Although the proven effectiveness and potential of carbon-based free-standing membranes are promising, the technology for the large-scale production of such membranes with adequate mechanical strength is still under development.

### 4.2. Surface Modification of the Existing Membrane Made from Other Materials

Pore wetting and fouling are the two significant issues with membranes, impacting the separation efficiency by allowing feed water to penetrate. Membranes with a charge similar to foulants can reduce fouling through electrostatic repulsion, which hinders the foulant from depositing on the membrane. Modifying the surface of polymer membranes has resulted in various low-fouling options. Employing nanoparticles might be an effective approach to creating membranes with lower fouling tendencies [55]. Surfaces with a high hydrophobicity have been shown to diminish the interaction between the membrane surface and the feed solution. These types of surfaces can be created through surface-modification techniques that employ various hydrophobic nanoparticles. The NPs are coated or grafted on the surface of the membrane that is being prepared. These membranes further developed additional mechanical strength and roughness [17]. CNTs coated on a polymer membrane do not have strong adhesion to the polymer matrix. The challenge remains of how to immobilize CNTs on the membrane surface without them being carried away. Moreover, the unique electrical properties of CNTs and graphene can be utilized to develop nanocomposite conductive membranes which exhibit an electrical repulsion effect. Upon their integration into MD membranes, the conductive channels provided by CNTs or graphene facilitate effective electron movement through the membrane. Applying voltage induces an electric field via the embedded NPs at the membrane’s surface which repels foulants carrying opposite charges, thereby inhibiting their attachment. Additionally, this increased electrical conductivity boosts heat and mass transfer, enhancing the flux rates and overall membrane performance. Nonetheless, an overly high voltage can inadvertently lead to electrolysis [3]. Kim et al. synthesized a hybrid membrane by coating single-walled CNTs (SWCNT) onto a PVDF membrane via a filter coating process and observed an improved conductivity without any loss of MD performance, even with a saline feed containing a high concentration of foulant [56]. Rao et al. reported the almost 100% elimination of the CaSO_4_ and silicate scaling using electrically conducting MD (ECMD) at low alternating potentials of 2 V. The ECMD membrane was synthesized by spray-coating CNTs onto the porous PP support and crosslinked by polyvinyl alcohol (PVA) [57].

### 4.3. Incorporation of the Carbon-Based Nanomaterials into the Membrane Matrix Forming Mixed Matrix Membranes (MMMs)

**Figure 3:**
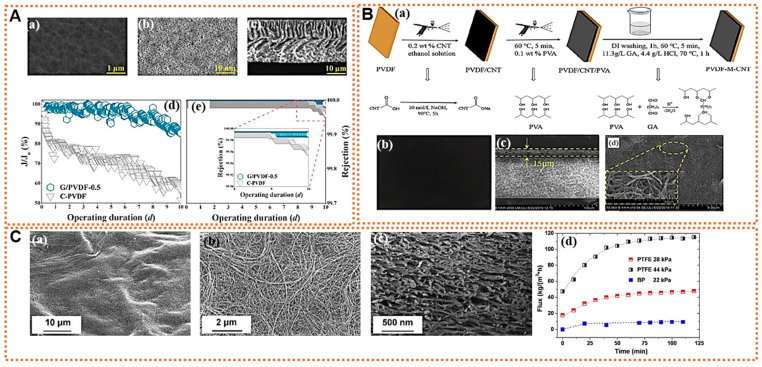
Different methods of incorporating carbon nanomaterials in/onto the membrane matrix; (**A**) SEM of graphene/PVDF-0.5 MMM prepared by phase inversion method, (**a**–**c**) are the top surface, bottom surface, and cross-section of the membrane, respectively. The appropriate 0.5 wt% loading of graphene showed larger and straighter finger-like pores with the highest porosity and pore size. As compared to commercial PVDF, the graphene-modified membrane exhibited excellent stability with almost constant flux and salt rejection up to 6 days of operation (**d**,**e**). Reproduced with permission from [62]. (**B**) Schematic illustration of the fabrication of the surface-modified PVDF-M-CNTs membrane (**a**), photographs of the top surface (**b**), and SEM micrographs of cross-section and top surface of the CNT-modified membrane (**c**,**d**). The modified membrane strip was densely covered by a layer of fibrous CNTs, giving a much less porous surface as compared to pristine PVDF membrane. Reproduced with permission from [63]. (**C**) SEM images of self-supporting BP membrane surfaces at different tilt angles (**a**,**b**), the cross section (**c**), and the flux variation comparison between PTFE and BP membranes as a function of time (**d**). Reproduced with permission from [52]. Image of surface shows the mat-like structure formed by randomly entangled CNTs while the cross section reveals the layered structure.

Low surface energy polymer can only bring about a limited hydrophobicity for practical application in MD. Incorporating carbon-based nanoparticles, such as carbon molecular sieves, CNTs, and GO, into the host polymer solution results in a MMM that improves hydrophobicity and pore anti-wetting characteristics, and also has the potential to modify the polymer’s free volume by changing the molecular arrangement of the polymer chains within the membrane [55]. As depicted in Figure 3, as an example, MMM formed by different methods incorporating different carbon nanomaterials showed different structures with different pore sizes and porosities that ultimately affected the membrane performances. Zahirifar et al. synthesized an octadecyl amine (ODA) functionalized GO/PVDF dual-layer flat sheet membrane. This modified membrane exhibited excellent stability and salt rejection via MD. This enhancement was attributed to the existence of GO-ODA on the top of the modified membrane that formed interconnected nano-channels with a high surface area, resulting in the high rejection of sodium chloride (NaCl). Similarly, the low thermal conductivity and high hydrophobicity further contributed to reducing pore wetting, temperature polarization, and heat diffusion across the membrane. The incorporated GO NPs also create a nanocapillary effect, allowing the selective sorption of water vapors [58]. Jafari et al. demonstrated that graphene quantum dots (GQDs) embedded in a PVDF nanofibrous membrane significantly enhance the water vapor flux and salt rejection during the AGMD process. The diminutive size of the GQDs promotes a more even particle distribution within the polymer solution, leading to the formation of a membrane with a more uniform structure. This improved membrane exhibited a modest reduction in its water contact angle and a notable increase in the LEP of water [59]. Similarly, Woo et al. have shown that graphene-enhanced electro-spun nanofiber membranes are highly effective in desalination through AGMD. They propose that the integration of graphene into the electro-spun polymer mixture results in membranes with a superior hydrophobicity, porosity, and surface-area-to-volume ratio, along with an interconnected pore network. Additionally, the nanofibers’ overlapping configuration contributes to a rough nanoscale texture, further augmenting hydrophobicity, which is beneficial for the MD process [60]. Likewise, their inherent hydrophobic characteristics and distinctive non-frictional transport capabilities make raw CNTs an excellent nanofiller for constructing MD membranes. Their large surface area and nanoscopic architecture serve as an efficient adsorbent for gases, which aids in the permeation of vapor molecules through the membrane. Moreover, their tubular structure offers an extra route for selective vapor transmission [61]. Eynolghasi et al. synthesized polystyrene/CNT MMMs via a phase inversion method and observed significant improvement in water flux with the increased porosity and average pore size. An optimum concentration of CNTs can give better results. However, the aggregation of CNTs in the membrane structure cannot prevent the passage of the salt effectively and cannot effectively transport vapor [61].

### 4.4. 3D-Printed Membrane Components for MD

**Figure 4:**
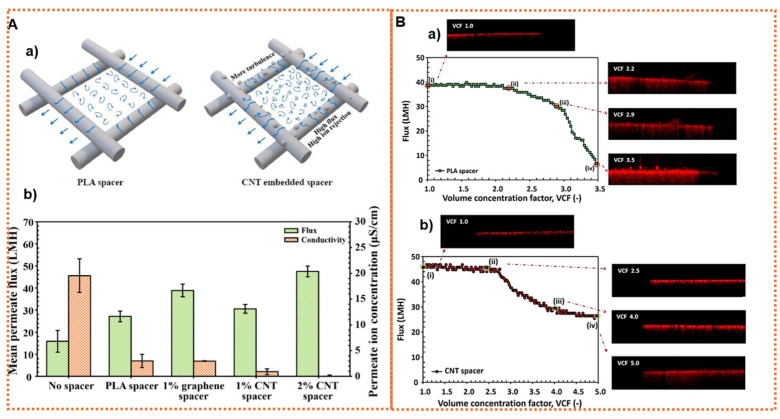
(**A**) (**a**) Schematic showing the flow pattern on 3D-printed PLA spacer and CNTs, embedded spacer, (**b**) graph indicating the effect of the different spacers on mean permeate flux (MPF) and ion rejection efficiency (IRE) in terms of permeate ion concentration. The order of MPF was as follows; (2%) CNT spacer > (1%) graphene spacer > (2%) graphene spacer > PLA spacer. IRE was above 97% when spacers were used. Reproduced with permission from [67]. (**B**) Optical coherence tomography images presenting the time-dependent scaling monitoring of the cross section of the 3D-printed membrane; (**a**) with PLA spacer, (**b**) with CNT spacer. PLA spacer membrane shows the scaling layer even at the volume concentration factor of 2.2, whereas CNT spacer membrane scaling was not observed even at higher volume concentration factor of 4.0. Reproduced with permission from [69].

Due to the flexibility of the geometrical design allowed by 3D printing technology, its potential in membrane preparation in MD is also gaining interest. This method paves the way for fabricating not only membranes but also feed spacers, offering high versatility and complex geometry. It incorporates nanomaterials to improve solar absorption and energy efficiency, which is particularly beneficial in solar-thermal desalination. The 3D printing technique enables the production of tailor-made membranes and module components with diverse shapes, enhancing the overall efficiency and allowing superior antifouling properties in the MD process. Kyoungjin An et al. introduced a 3D superhydrophobic CNT-PVDF-hexafluoropropylene (HFP) composite membrane fabricated by electrospinning and showed its improved efficiency in the MD process. They demonstrated that CNTs enhanced the repulsion force in Knudsen molecular diffusion, which enabled rapid vapor transport while maintaining anti-wetting characteristics [64]. Similarly, as vapor permeates through the pores of the MD membranes, it creates a higher local supersaturation level close to the membrane’s surface, leading to surface crystallization. However, this can be mitigated by enhancing the turbulence and mixing at the membrane surface with an appropriate feed spacer geometry, thereby altering the hydrodynamic flow conditions to decrease membrane surface crystallization [65]. Utilizing 3D printing technology, a functional spacer can serve as a photothermal surface for heating in the MD process. By positioning these spacers on top of membranes, they can create turbulence flows that enhance the solar absorption and reduce fouling. A key focus is on designing photothermal feed spacers that support surface heating in MD through a localized photothermal effect generated solely by the spacers near the distillation membrane’s surface [66]. Spacers printed with the integration of carbon-based nanomaterials can achieve a photothermal conversion effect that generates heat on the surface of the distillation membranes when exposed to sunlight. CNTs and graphene are particularly promising as additives in these spacers due to their thermal and electrical conductivities, along with their robust mechanical strengths. Incorporating such nanofillers not only significantly enhances the properties of the otherwise fragile plastic spacers but also diminishes the polarization effect, leading to an enhanced performance of the MD process [67]. Using a 3D printing technique, as depicted in Figure 4A, Jeong et al. created spacers embedded with CNTs/graphene nanoparticles, resulting in a significant increase in the permeating flux compared to the control made of polylactic acid (PLA). This increase in flux is attributed to the enhanced local turbulence caused by the multiscale roughness on the spacer’s surface, which, in turn accelerates the vaporization rate across the membrane [67]. Likewise, the building up of CaSO_4_ and CaCO_3_ scales poses a significant challenge in MD. The formation of such scaling on the membrane surface can exacerbate the adverse effects of temperature polarization and concentration polarization, leading to a reduction in the membrane’s active area for water evaporation and, as a result, a lower distillate flow [68]. Jeong et al. further studied the scaling control mechanism of using carbon nanomaterials in MD, developing 3D-printed CNT spacers. As shown in Figure 4B, using an optical coherence tomography technique, they observed that increasing the spacer roughness by introducing CNTs enhances the turbulence, reduces the surface scaling on the membrane, and, hence, improves the MD efficiency [69].

## 5. Major Carbon-Based Nanomaterials in MD Process

The function of carbon-based nanocomposite membranes in MD goes beyond enhancing the physical and chemical properties of the membranes and the overall efficacy of the process. They have also opened the door to creating membranes with distinctive features like self-heating and self-cleaning capabilities. Membranes which incorporate CNTs and graphene are considered as enhanced membranes due to their superior thermal and chemical stability, increased flux, better salt rejection, and fouling resistance.

### 5.1. Carbon Nanotubes (CNTs)

**Figure 5:**
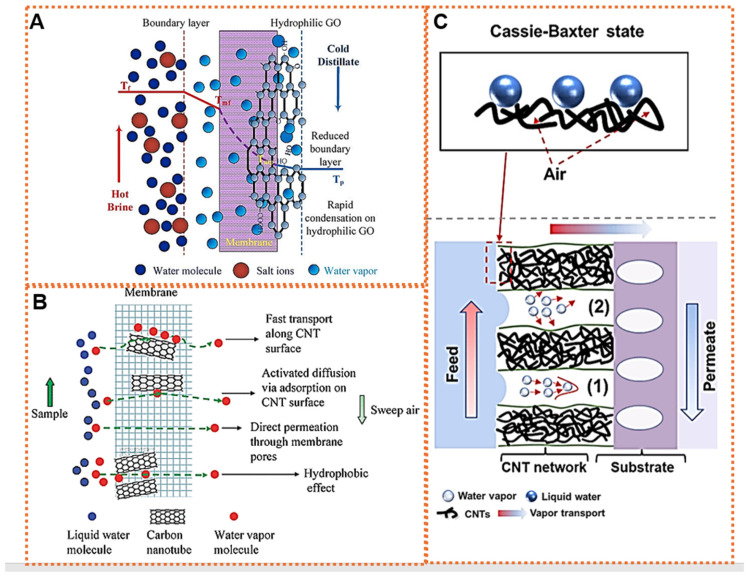
(**A**) Schematic showing the proposed mechanism of vapor transport in membrane modified with GO in permeate side in MD process. Due to incorporation of GO in the permeate side, a boundary layer compromising both liquid and vapor phase is formed on both sides of the membrane, permitting fast water vapor removal, destabilization of the vapor-gas, and reduction in mass transfer resistance between the bulk permeate and membrane surface. Reproduced with permission from [94]. (**B**) Mechanism of MD in presence of CNTs. Immobilization of CNTs in the pores alters the vapor transport rate via multiple pathways such as fast transport along the CNTs’ surface, activated diffusion via adsorption on the CNTs’ surface, direct permeation through membranes pores, and hydrophobic effect. Reproduced with permission from [95]. (**C**) Water wetting behavior (Cassie–Baxter model) when placed onto the superhydrophobic CNTs network membrane surface (upper) and codominant mechanism of enhanced water-vapor transport across the CNT network membrane; (1) viscous flow and (2) Knudsen diffusion (lower). Based on dusty gas model, the mechanism of water-vapor transport through CNT membrane pores was dominated by the combined mechanism of Knudsen diffusion and viscous flow. Reproduced with permission from [85].

The incorporation of CNTs into advanced membrane separation technologies stands at the forefront of innovative research within both membrane technology and materials science, representing significant developments in various carbon-based nanomaterials used in water treatment. CNTs are smooth, rolled-up graphitic sheets which create a tubular network of bent sp^2^ hybridized atoms. These nanomaterials are characterized as a one-dimensional material due to their very high aspect ratio [70]. Their outstanding physical and chemical properties, such as their hydrophobicity, large surface area, interconnected open pores and layered structure, good thermal, mechanical and chemical stabilities, and tunable chemistry, have attracted CNTs as a potential nanomaterial for separation applications including MD [71,72]. As shown in Figure 5B, the immobilization of CNTs within the pores of a hydrophobic membrane has been shown to effectively modify the interactions between water and the membrane, thereby enhancing the vapor permeability and inhibiting the filtration of liquid into the membrane pores. Choi et al. were the first to attempt to apply CNTs to membrane preparation [73]. The excellent fluid transport rate and the fast size-exclusion selectivity feature of CNT-based membranes enable promising applications, including water purification/water desalination [74]. Nevertheless, integrating unmodified CNTs into the membrane matrix faced challenges due to the CNTs’ weak interfacial compatibility with the polymer, resulting in non-selective voids within the membrane [75]. Likewise, creating uniform nanocomposite membranes that contain a high amount of CNTs remains challenging due to the poor dispersibility of CNTs, which complicates their chemical application [76]. To reduce the Van der Waals forces between CNTs and ensure their even dispersion in the polymer matrix, it is necessary to modify the CNTs with specific functional groups. It was suggested that the functionalization of CNTs using carboxylic acid [77], poly (sodium 4-styrenesulfonate) (PSS) [78], and inorganic materials like titanium dioxide (TiO_2_) [79] and ferro ferric oxide (Fe_3_O_4_) [80] could provide a good reaction with the polymers.

Research suggests that water molecules pass through CNTs at a rate of 1000–10,000 times faster than the Hagen–Poiseuille equation would predict [81]. This rapid movement, known as the slippage effect, occurs due to the smooth hydrophobic walls of CNTs allowing water to flow without friction. A surface with higher hydrophobicity promotes not only the removal of water from the outer surface of the CNTs, but also facilitates the rapid and frictionless transport of water vapor. As illustrated in Figure 5C, the CNT network layer provides the membranes with high salt rejection and a promising water flux. This improved flux was attributed to an extremely low liquid–solid contact interface, enabling water vapor to swiftly transverse the membrane structure through a combination of Knudsen diffusion and viscous flow, while achieving an effectively high-salinity feed by establishing a Cassie–Baxter state.

Majumder et al. observed water flow rates between 9.5 and 43.0 cms^−1^bar^−1^ through a VA-CNT membrane with 7 nm pores and a slip length ranging from 39 to 68 nm [82]. Molecular simulations have shown that water molecules move more quickly through CNTs with a narrower inner diameter [83]. Si et al. developed a networked functional layer of CNTs on a stainless-steel substrate, showcasing a superhydrophobic and super porous triple-phase interface that enhances the potential of MD applications for treating high-salinity water [84]. Likewise, Sun et al. investigated the high-flux transport mechanism at the molecular level through a membrane structure that was designated in a rational manner. This study utilized experimental methods as well as molecular dynamics simulations [85]. The research involved the design and preparation of a stainless-steel substrate with a highly controllable structure, which includes an adjustable sponge-like region and finger-like micro-voids. This was achieved by optimizing dry–wet spinning parameters such as the solid-state loading, pore air flow, and air gap distance. Subsequently, CNTs were grown in situ on the stainless-steel membrane substrate using a self-catalysis chemical vapor deposition (CVD) process. When tested with simulated seawater (35 gL^−1^ NaCl) in a VMD system, the CNT network membrane exhibited a high salt rejection rate (>99%) and a high-water flux (43.2 LMH) for 2 h. However, there was a slight decrease in the flux after 12 h of operation due to membrane corrosion. The researchers suggested that the ultra-low fraction of the liquid–solid contact interface (<3.23%) facilitated rapid water-vapor transport across the superhydrophobic, super porous membrane network structure through Knudsen diffusion (molecule–wall collision) and the Cassie–Baber model (i.e., excellent anti-wetting ability). In another study, Dong et al. further regulated the two different kinds of CNT-incorporated membrane structures, PC-CNT (partially covered) and FC-CNT (fully covered) and studied their separation performances in the DCMD system [71]. The FC-CNT membrane was observed to maintain a nearly constant flux of 37.1 LMH, exhibit excellent salt rejection of over 99.9%, and demonstrate an improved LEP of 2.0 bar. The LEP value is sufficiently high to impede water permeation during DCMD. In contrast, the PC-CNT membrane displayed a high-water flux of 41.1 LMH but had low salt rejection and a low LEP of only 0.6 bar, which allowed membrane wetting to occur. The exceptional performance of the FC-CNT membrane can be attributed to its high anti-wetting behavior and unique membrane structure. The membrane consists of a super porous and superhydrophobic surface layer composed of CNTs, resulting in a large liquid–vapor interface between the feed and the membrane. Additionally, the interior of the membrane features long-channel fingerlike macro-voids decorated with CNTs, further enhancing its performance. Wang et al. proposed a robust super-hydrophobization process sandwiching a CNT layer over commercial PVDF, indirectly grafting the low-surface-energy materials 1H, 1H, 2H, and 2H-perfluorodecyltriethoxysilane (FAS) [86]. The synthesized membrane with a high coated angle (180°) maintained hydrophobicity even in harsh conditions and showed no decline in flux throughout the DCMD process. The improved flux of the CNT-sandwiched membrane as compared to the bare membrane was attributed to an improved heat–mass transfer, contributed to by the CNT layer via facilitated heat conduction and vapor accumulation within the CNT network. Kyoungjin An et al. incorporated functionalized CNTs into nano fibers (PVDF-HFP polymer) of the electro spun membranes and evaluated the MD efficiency in the DCMD system using NaCl as feed stock [64]. They made an observation that the inclusion of CNTs resulted in a more rapid vapor transport across the membrane compared to when CNTs were not present. They proposed that the superhydrophobic nature of CNTs enhances the repulsive energy between the pore and vapor, thereby increasing the Knudsen and molecular diffusion coefficients. This, in turn, reduces the boundary layer effect and promotes the faster flow of viscous fluid through the membrane’s pores. Additionally, the formation of beads caused by the clustered CNTs in the nanofibers contributes to an increased overall surface roughness of the membrane, ultimately improving vapor transport. Fransis et al. used an electro-hydrodynamic atomization technique to uniformly deposit CNTs onto commercially available PTFE membrane and showed a superior desalination performance, with a constant water-vapor flux for 24 h of continuous DCMD operation and a 99.99% rejection of the inorganic salt compared to PTFE membrane [87]. Electrospray deposition enables the creation of micro-patterns of CNTs on a PTFE membrane. This process induces micro-turbulence in the solution surrounding the membrane surface, resulting in an elevated temperature polarization coefficient. Consequently, the propensity for salt deposition during the DCMD operation is diminished. Xie et al. used a facile one-step spray coating technique and fabricated a CNT-enhanced MD membrane. They proposed enhancing the specific surface area of the substrate membrane, which increases the vaporization surface area and leads to improved flux during the DCMD process. Furthermore, when examining membranes with comparable properties, the arrangement of CNTS coated on the substrate membranes can be ranked as PP < PVDF < PTFE to attain the highest specific surface area for the membrane. Additionally, the thermally conductive properties of CNTs can effectively mitigate temperature polarization on the membrane surface, thereby improving the MD process. It is also crucial to optimize the amount of coating, as an excessively high coating causes a reduction in the pore size of the membranes, consequently increasing the resistance to mass transfer [88]. Bhadra et al. designed a free-standing CNT membranes using carboxyl (COOH) functionalized multiwalled CNTs (MWCNTs). These membranes show high polarity and high interaction between the membrane surface and the water vapor, enhancing the MD process [89]. Dumée et al. modified CNTs into BP structures, creating a self-supporting membrane with improved hydrophobicity via grafting alkoxysilane. The alkoxysilane-modified CNT BP exhibited a better performance and an increased lifespan by 50% more than the non-treated samples. The LEP was increased from 193 kPa to 441 kPa in the silanised CNT BP. The higher LEP was correlated to the improvement of hydrophobicity. Increasing hydrophobicity enhances the flux because it enlarges the water meniscus surface, thereby providing more exchange surface for water evaporation [90,91]. In a separate study, Miao et al. introduced the MD device, which incorporated CNTs as a solar absorption layer, a qualitative filter paper as a water transmission pipeline, and aerogel blankets as thermal insulators. This innovative design aimed to optimize solar harvesting and heat localization for improved water evaporation. The researchers successfully achieved a remarkable thermal conversion efficiency of up to 84% under a light intensity of 1 kWm^2^. Additionally, the MD device exhibited exceptional cyclic stability, maintaining its performance over 10 cycles [92]. Similarly, Huang et al. introduced a groundbreaking technique called electric field-assisted vacuum MD (EVMD). This method employed either MWCNTs or a combination of MWCNTs and graphene as a conductive substrate, with PTFE serving as the base membrane. By forming a nanostructure on the membrane, consisting of graphene/MWCNTs, the hydrophobicity of the membrane was enhanced, leading to an improvement in the distillation flux. Additionally, MWCNTs/graphene exhibited exceptional thermal conductivities, which helped alleviate temperature polarization and further prevent a decline in the MD flux. The application of EVMD effectively addressed membrane fouling by leveraging the electric-field and electrochemical effects against pollutants. Notably, this membrane demonstrated superior antifouling properties when subjected to intermittent electric fields with a field strength of 1.0 Vcm^−1^ [93].

### 5.2. Graphene Oxide (GO) and Reduced Graphene Oxide (rGO)

GO coating has garnered attention in the water treatment process due to its exceptional properties resulting from its structured honeycomb lattice with oxygen-rich functional groups. With excellent dispersibility and the ability for scalable production, GO has been widely utilized in the MD process. It can be used either as a standalone component in the form of a laminate or integrated into other polymers to produce MMMs. It exhibits better miscibility with polymers and superior mechanical stability [42]. GO laminates have a higher thermal conductivity of 20 Wm^−1^K^−1^, which surpasses that of PTFE material. This disparity in thermal conductivity enables GO laminates to effectively diminish the heat transfer resistance of the boundary layer. Consequently, this reduction in resistance leads to an elevation in the temperature of the membrane surface [96]. Furthermore, the efficient removal of water vapor from the permeated-side boundary layer is also crucial to elevating the concentration gradient to improve mass-transfer efficiency. The schematic in Figure 5A suggests the reduction of the boundary layer because of membrane modification in the permeated side. This ultimately improves mass-transfer efficiency.

Due to its extensive functionalities in the GO, it can also be effortlessly functionalized with additional molecules to alter its characteristics [97]. Holmes et al. created a PVDF/Y-aminopropyl triethoxysilane (APTS)-functionalized GO MMM, resulting in an 86% increase in permeated flux compared to PVDF, while still maintaining over 99% salt rejection in the AGMD process. The enhancements were due to a higher surface and bulk porosity, larger mean pore size, and beneficial water–nano-filler interactions facilitated by the functional groups of GO-ATP [98]. In their study, Wen et al. found that a GO membrane modified with perfluoroalkylsilanes (PFAS) displayed improved selectivity in hydrogen isotopic water separation compared to commercial membranes in the AGMD process. They achieved a maximum mean separation factor of 1.067 along with a permeating flux of 0.47 kgm^−2^h^−1^. The enhanced separation factor in membranes with a large flake size indicated effective molecular sieving properties for isotopic water molecules within nano channels. This enhancement was attributed to the efficient collaboration between unobstructed nano channels and the nearly superhydrophobic surface [99]. Xu et al. investigated the impact of different oxygenic group contents of GO at varying oxidation temperatures on the MD performance. The PVDF membrane’s permeating side was coated with modified GO during the DCMD process. The rejection of solute by the GO layer prevented the decline in permeation quality due to membrane wetting. The flux enhancement was more pronounced in GO with fewer OH groups and more epoxy groups. Converting OH groups to epoxy groups in GO was found to be an effective strategy for enhancing the water flux [100]. Likewise, Seo et al. introduced a chemical vapor-deposited (CVD) graphene membrane featuring nano channels that allow the passage of water vapor. These nano channels, formed through the mismatching of the overlapping of graphene domains, effectively decrease the flow resistance, enabling rapid water-vapor transport. Despite being mildly hydrophobic (with a contact angle of 81.3°), these membranes exhibit superior anti-wetting and antifouling properties compared to highly hydrophobic PTFE (with a contact angle of 131.3°). The interaction between graphene and contaminants is characterized by weak physisorption [101]. In a similar vein, the rGO/Nafion/Ni foam 3D network structure was introduced by Pan et al. This structure exhibits an effective induction-heating mechanism, resulting in a sustainable and exceptional performance for MD. The enhanced absorption of electromagnetic waves through multiple internal reflections and the generation of larger eddy currents by rGO contribute to an elevated temperature at the feed membrane interface. This, in turn, amplifies the driving force for distillation, ultimately enhancing the MD efficiency [102].

### 5.3. Carbon Black (CB)

CB, a carbonaceous material, shares the same chemical backbone as most organic compounds, making it hydrophobic. This characteristic has led to the use of low-cost CB in creating superhydrophobic membranes. To further enhance its properties, CB can be modified by hydrophobic silane being incorporated into the polymer membrane. The silane coating serves to decrease the surface energy of the solid surfaces, resulting in superhydrophobic behavior. Additionally, CB is employed to improve the mechanical strength of the membrane. The silane-modified membrane displays a significantly higher level of superhydrophobicity, boasting a contact angle of 160°, which, in turn, leads to an increased water flux and superior salt rejection (>99.9%) [103]. Zakaria et al. utilized CB to enhance the hydrophobic properties of the PVDF membrane using 3D printing technology. Their findings revealed that the inclusion of CB in the casting solution led to a reduction in finger-like voids, an increase in the membrane thickness due to slower phase separation, and a decrease in the pore size and porosity with a higher CB concentration, causing pore blockage. The introduction of CB particles also resulted in an increase in surface roughness, creating a superhydrophobic surface even without the presence of additional hydrophobic agents. Interestingly, the permeated flux of the modified membrane remained consistent with that of the original PVDF membrane. One notable advantage of this modification was the ability to electrochemically clean the membrane in just 4 min, restoring its permeated flux after exposure to a salt solution containing surfactants [104]. Chem et al. created a dual-functional, omniphobic–photothermal nanocomposite membrane by utilizing a hierarchical structure of FAS17 modified with CB NPs on a PVDF substrate. This innovative membrane exhibited excellent resistance to wetting and required minimal energy consumption during desalination through the direct solar MD process. The fluorinated CB NPs absorbed sunlight, generating localized heating that increased the membrane flux by 25%. Additionally, the re-entrant structure formed by CB NPs in conjunction with the hydrophobic FAS 17 coating resulted in a low surface energy, thus achieving omniphobic properties [105]. Table 1 gives an overview of the different polymers and carbon nanoparticles used, their incorporation technique used and applications in different MD process as well as their performance efficiency.

## 6. Carbon-Based Nanocomposite for Photothermal/Joules-Heating MD

**Figure 6:**
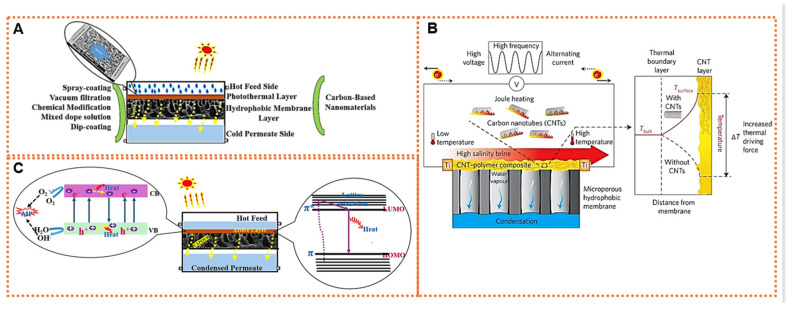
(**A**) Schematic of direct heating of membrane surface in MD using deposited carbon-based NPs on a hydrophobic membrane surface. Inset is the CNT-based flat sheet membrane for MD. Reproduced with permission from [130]. (**B**) CNT-based joule heating membrane for MD. Reproduced with permission from [127]. (**C**) Schematic showing mechanism of photocatalytic process coupled with MD. Inset is the schematics showing working principles of carbon-based photo-thermal membrane for MD. Inset on left shows the electron-hole generation and relaxation mechanism while the right shows molecular thermal vibration mechanism. Reproduced with permission from [117].

The complexity of the system and the potential loss of thermal energy are increased when the feed is heated externally in conventional MD. A significant drawback of the conventional MD process is the temperature polarization phenomenon, which reduces the effective temperature difference across the membrane. The polarization occurs on both sides of the membrane and leads to a decrease in vapor pressure difference, ultimately hindering the practical application of the membrane [113]. Surface-localized heating through the utilization of solar energy or electric power is an innovative approach to combat temperature polarization, which serves as the main cause of thermal inefficiency in traditional MD systems. This technique significantly reduces energy consumption for both heating and circulating purposes [114]. In this method, the feed is heated directly at the surface of the membrane, eliminating the need for pre-heating outside the membrane module. The feed solution can be heated at the membrane surface through either electro-thermal or photothermal processes. The key components of electro-thermal MD are conductive materials and the resulting membranes, which consist of two main elements: conductive polymer or nanomaterials that provide electrical conductivity, and a hydrophobic porous membrane layer that offers structural support and a pathway for vapor transport. Adding an extra layer with photoactive materials onto the surface of commercially available or synthesized polymeric membranes is considered an effective approach [115]. The schematic in Figure 6A illustrates the MD process employing the direct heating of the hydrophobic membrane surface, which has been modified by the inclusion of various carbon-based NPs and using different deposition methods.

The layer of photoactive coating can supply consistent heat to ensure uniform thermal propulsion throughout the membrane, thus overcoming temperature polarization [116]. An approach to lowering the energy expenses of MD processes involves the utilization of solar energy, a method that necessitates both technical and economic validation. A novel photothermal MD (PMD) technique has been developed to capitalize on the plentiful solar energy that is available and concentrate heating at the interface of the membrane and feed water by leveraging photothermal effects. This phenomenon is triggered by photoexcitation, leading to the generation of partial or complete thermal energy. By focusing on the membrane–feed water interface, this method eliminates the need to heat the entire bulk of the feed water and transport feed from heat sources to membrane modules, resulting in significant energy savings. Furthermore, this approach eliminates the need for complex equipment and power-generation systems [117]. PMD introduces materials with the ability to convert light energy into heat in the distillation process, allowing for precise control. The nanostructure’s material-induced photothermal effect offers the advantage of precise heat modulation in a specific region at the nanoscale. By utilizing photothermal materials to absorb solar radiation, PMD efficiently generates localized temperature elevation, driving the evaporation and phase transition processes. These photothermal active layers also provide localized heating at the evaporation surface, enhancing the vapor-permeation driving force and reducing the energy required for heating the feed. The PMD system involves three main conversion processes: (1) the photothermal process, which converts light to heat through the photothermal effect of nanomaterials; (2) the vaporization process, which transforms water into vapor phase using the generated heat; and (3) the condensation process, which converts vapor into distillate based on various condenser designs. Therefore, when designing the PMD system, both the solar-to-thermal conversion capability of the photothermal nanomaterials and the characteristics of the PMD configuration contribute to its overall performance [118,119].

Efficient light utilization requires the use of photo-absorbers that have strong absorption and a high photothermal conversion efficiency. Carbon-based materials, such as CNTs, carbon nanostructures, graphene, and rGO, have been extensively adopted as photothermal materials due to their high abundance, scalability, low cost, ability to absorb the full solar spectrum, and stability. These carbon-based nanomaterials have been widely studied in the fabrication of electrically conductive membranes because of their excellent conductivity, uniform heating, and hydrophobicity. Additionally, the ability of photothermal materials to absorb solar radiation plays a crucial role in determining the amount of heat energy that can be converted from light energy. In this regard, carbon nanomaterials are naturally black, making them suitable for broadband solar absorption [120]. The efficiency of converting sunlight into thermal energy plays a crucial role in enhancing the overall performance of photothermal systems. This conversion process relies on the molecular vibrations induced by the absorbed sunlight. By matching the photon energy of incident light with the electron transitions in the molecules, carbon-based materials can excite electrons from the ground state (HOMO) to a higher energy state (LUMO) (π-π*), causing them to release heat when they relax back to the ground state [117,121]. Various carbon-based absorbers like GO, CB, graphite, carbon composites, CNTs, and amorphous carbons exhibit exceptional solar-to-heat conversion capabilities due to their unique properties. These materials can float on water surfaces, thanks to their tunable low density and hydrophobic nature, providing insulation to minimize heat loss. The absorbed sunlight generates a power field in photothermal materials, energizing mobile carriers within the crystal lattice and converting their energy into heat [122,123]. Numerous meticulously engineered nanostructures made of porous carbon have been created with the aim of achieving optimal light absorption for stem generation. The porous configuration can be tailored using either one-dimensional (1D) or two-dimensional (2D) components, such as an array of 1D carbon nanotubes (CNTs), a vertically aligned 2D sheet of graphene, or a graphene foam composed of interconnected graphene sheets forming a porous network structure that exhibits a blackbody-like property [124]. CNTs exhibit a thermal conductivity of approximately 1000 Wm^−1^K^−1^, while GO nanoparticles demonstrate a significantly higher thermal conductivity of around 5000 Wm^−1^K^−1^. The unique structure of graphene provides water vapor sorption sites within its hexagonal honeycomb lattices, which consist of sp^2^-bonded carbon atoms along with polar functional groups such as OH and COOH. These features facilitate enhanced interactions between the membrane and water vapor, thereby increasing the water vapor flux. By applying a layer of such carbon nanoparticles onto polymeric membranes, the thermal conductivity across the membrane surface can be improved without affecting the overall thermal conductivity of the bulk membrane, resulting in decreased heat loss [125]. The carbon-based nanomaterials in the photothermal MD system’s active layers generate localized heating on the evaporation surface, thereby increasing the driving force of vapor permeation and consequently lowering the energy needed to heat the feed [118]. Lu et al. have developed an ultrathin membrane based on PVA hydrogel which exhibits a unique combination of facilitated vapor transfer and environmental energy harvesting for solar-driven MD. This innovative membrane design incorporates a hydrogel-evaporating layer that possesses both water-proof properties and a high solar-vapor-generation rate. As a result, the vapor collection ratio surpasses 80%, leading to a remarkable water yield of 2.4 kgm^−2^h^−1^ under one sun irradiation, without the need for energy recycling or cooling accessories. The inclusion of a CB layer in the membrane allows for the conversion of solar irradiation into heat, which in turn powers the water evaporation process. Furthermore, the porous structure of the CB layer facilitates the rapid transfer of water to the hydrogel layer through the capillary action [126].

The photothermal approach to self-heating membranes faces significant limitations, primarily due to the challenge of effectively irradiating a large surface area using UV or solar irradiation. This limitation restricts its application to small-scale MD processes. Additionally, not all photothermal materials used for coating can be exposed to light, and an excessive loading of these materials can lead to pore blockage in the membrane, resulting in a reduced distillation flux. To ensure the long-term and efficient operation of solar self-heated MD systems with improved productivity, membranes coated with high-photothermal-conversion materials must be employed [127]. Likewise, achieving consistent freshwater production without the support of an auxiliary heating system or heat storage facilities proves to be challenging due to the fluctuating intensity of sunlight under varying weather conditions and the time of the day. Prolonged severe weather conditions can hinder the sustainable operation of photothermal MD systems.

Electric current application, as a method of joule-heating, has been receiving significant interest. Known also as Ohmic or resistive heating, this method generates heat due to the resistance a conductor offers to electron movement. By directly heating the surface, it increases the vapor flow by raising the feed’s temperature at the membrane’s surface, thereby reducing temperature polarization’s impact. Joule heating enhances thermal energy efficiency and improves the output while consuming less specific energy. Any material utilized for joule heating should be electrically conductive and have adequate electrical resistance to facilitate resistivity heating [128]. Joule MD utilizes an innovative setup where an electro-thermal membrane directly warms the feed, eliminating the need to acquire external heat. Consequently, this reduces the energy required for hydraulic circulation and minimizes the heat dissipation in the process [129]. Carbon nanomaterials are strong choices for heat-related applications that do not use metals because they conduct heat very well, have great electrical traits, and convert electricity to heat efficiently. Out of all carbon-based nanoparticles, CNTs are especially good because they are not too expensive and can be used to make conductive, porous layers. These qualities make them perfect for joule heating elements in a thermally driven separation process. Figure 6B shows an overview of the joule heating operation utilizing the CNT-based membrane. CNTs can heat up salty water just by being on the membrane’s surface, which helps with the MD or desalination processes by providing the necessary heat without an outside source [127]. CNT-based joule heaters have also shown the ability to function effectively in low-ionizable conditions and non-corrosive environments. This means that, even when high-potential direct current is applied for resistive heating, the stability and heat transfer properties of the CNTs remain uncompromised [119,127]. In comparison to raising the bulk feed temperature, the use of assisted joule-heating has proven to enhance thermal energy efficiency and boost productivity while consuming less specific energy [128]. The employment of electro-thermal materials in ionizable surroundings leads to water splitting and materials’ deterioration, even at low potential charges on the surface, thereby restricting its extensive use. Dudchenko et al. developed a porous CNT-PVA joule heater and proved its capability of being heated directly in a highly corrosive and ionizable environment up to 20 V without experiencing notable performance decline. Their finding suggested that increasing the frequency of the applied alternating current to a certain extent can effectively safeguard the membrane from deterioration [130]. Since then, extensive research, using carbon nanomaterials, has been conducted on the joule-heating MD system. In order to enhance its energy efficiency, heat recovery devices can be integrated with the joule MD process. Huang et al. presented a joule-heating MD process that incorporates a self-heating composite membrane consisting of a polydimethylsiloxane (PDMS)/MWCNT/PVDF tri-layer along with a multi-level thermal concentration and multi-level heat recovery device. The maximum freshwater productivity achieved was 2.77 Kgm^−2^h^−1^, with an energy consumption of 0.36 KWhKg^−1^ [131]. Anvari et al. showcased a contactless induction heating MD technique employing a PTFE membrane coated with iron oxide-CNTs and subjected to a high-frequency magnetic field. Through this method, they achieved a remarkable freshwater production rate of 4.025 Kgm^−2^h^−1^ and a salt rejection exceeding 99% under a low vacuum condition of 20 kPa [132]. One significant drawback of joule-heating MD technology is its reliance on expensive high-grade electrical power, which cannot easily be replaced by renewable or waste heat sources. Therefore, an alternative approach to enhance the MD process involves integrating photothermal and joule-heating processes to elevate the temperature of the membrane surface during both daytime and nighttime MD operations. In this method, various carbon-based nanoparticles capable of absorbing solar radiation and converting it into heat were employed to modify the membrane surface. Huang et al. synthesized a PDMS/MWCNT/PVDF tri-layered composite membrane that provides photo-thermal and joule-heating properties, simultaneously allowing both photothermal and joule-heating MD separately or in combination [133]. Dongare et al. synthesized a bilayer-structured photothermal membrane central to nanophotonic-enabled solar MD (NESMD). A thin optically absorbing porous PVA was coated onto the PVDF membrane. The CB possessing the broadband absorption was dispersed into PVA solution prior to electro-spinning of PVA into PVDF. Fresnel lenses were used to concentrate sunlight by 25 times, which achieved a higher flux of 5.20 kgm^−2^h^−1^ compared to the unfocussed case (0.22 kgm^−2^h^−1^). They concluded that, unlike MD, this NESMD benefits from increases in scale and in ambient operating temperatures and requires only a modest flow for optimal distillation conversion [134]. Wu et al. fabricated a PVDF membrane by incorporating SiO_2_/Au nano-spheres and un-functionalized CB NPs, resulting in a membrane that exhibited an enhanced performance. The water flux was found to increase by up to 33% compared to the flux observed in the absence of light when using a bench-scale NESMD system operating in direct contact mode under simulated sunlight at one sun unit. The researchers demonstrated that the photothermal coating’s surface heating could enhance thermal efficiency, thereby reducing temperature polarization during the MD process [135]. Moreover, the combination of simultaneous photothermal conversion, the localized heating of feed water, and heat concentration near the membrane has the potential to enhance the performance of the MD even further. By integrating an alternating current and sunlight exposure into the membrane concurrently, the synergistic effects of photothermal and joule-heating MD can be harnessed within the same MD module, eliminating the need for extra support structures or equipment. This setup allows for the adjustment of the joule-heating power in response to fluctuations in the solar input power, ensuring that a consistent total input power level is maintained. Huang et al. synthesized a tri-layer composite membrane using bottom PVDF, middle MWCNT, and top PDMS. The MWCNT is the key to a membrane with a good photothermal and joule heating conversion ability. MWCNT has the dual property of converting sunlight to thermal energy and electrical energy to thermal energy at the same time, achieving a higher temperature than the stand-alone process and, thus, higher efficiency [133].

## 7. Photocatalytic MD Process

To address membrane fouling in MD applications, it is crucial to employ technologies with diverse functionalities, such as organic degradation and minimizing the attachment of inorganic and organic constituents to the membrane surface. An appealing method to achieve this is by modifying the membranes with photocatalytic semiconductor NPs, which can reduce the interaction between water constituents and the membranes. Upon exposure to light, these NPs can generate reactive oxygen species (ROS) that can decompose and potentially mineralize organic contaminants in wastewater [136]. The incorporation of photocatalytic nanoparticles into the membrane surface in the photocatalytic MD technology allows for the integration of the oxidation process with the MD. This integration leads to enhanced antifouling properties, decreased reliance on chemicals and reduced costs. Additionally, the extensive pretreatment required in traditional desalination processes is minimized [136]. Photocatalytic membranes have emerged as a highly effective antifouling strategy in MD. These membranes are photo-responsive and possess the ability to resist fouling while also facilitating the cleaning of the fouled membranes. This is achieved through the process of photocatalytic degradation, where contaminants are broken down by the generation of ROS when exposed to light. Importantly, this method ensures the complete degradation of pollutants without the production of any secondary pollutants [3]. Meanwhile, immobilizing photocatalysts onto the membrane not only offers a solution to the reusability issue of powdered photocatalysts but also establishes an efficient catalytic setup. Nevertheless, a significant issue lies in adjusting the naturally slower photocatalytic degradation process to align with the requirements of high-flux water purification in MD systems. Additionally, the polymer membrane in this configuration presents challenges due to its susceptibility to UV light and the potential breakdown of its structure caused by the oxidation impact of the reactive species produced [137]. The schematic in Figure 6C shows the mechanism of the photocatalytic process coupled with MD. Ceramic membranes, which exhibit superior mechanical, thermal, and chemical stability compared to polymers, and are capable of withstanding chemical damage caused by photocatalytic environments and physical stresses during operation, are highly favored. Nevertheless, their utilization remains constrained due to the significant expenses associated with their production [138]. Similarly, ceramic membranes typically exhibit hydrophilic characteristics, necessitating surface modification to confer hydrophobic properties for their application in the MD process [139]. Research on combined photocatalytic MD is limited. Chen et al. synthesized the photocatalytic MD process using a TiO_2_-coated ceramic membrane and found that this photocatalytic MD achieved a high removal of >99.99% of metals and 89.7% of organics from the produced water. This MD method significantly reduced fouling and maintained a stable operation of produced water treatment [136]. Szymarnski et al. devised a submerged photocatalytic membrane reactor that effectively combined photocatalysis with DCMD. To achieve this, they utilized TiO_2_ photocatalytic nanoparticles which were suspended in a feed solution. The experimental findings demonstrated a remarkable outcome, as the ketoprofen compound underwent nearly complete photo-oxidation. This was evident from the absence of any detectable ketoprofen in the distillate produced by the reactor [140]. The utilization of TiO_2_ as a UV active photocatalyst has been widely reported in most photocatalytic MD processes. However, there is a growing interest in the development of visible light-driven systems, which offers new possibilities for achieving high-performance photocatalytic MD. In this regard, carbon-based NPs have emerged as a promising alternative to plasmonic metals and semiconductors. Carbon-based NPs possess several advantages such as abundance, scalability, low cost, and excellent stability. Among these, 2D materials have garnered significant attention in photocatalytic MD processes due to their abundant active sites’ conductivity and chemical stability. One such material is graphitic carbon nitride (g-C_3_N_4_), which is a metal-free photocatalyst with customizable structures and remarkable stability. g-C_3_N_4_ has attracted considerable interest for its potential applications in photocatalytic membrane preparation. Its unique combination of inorganic and organic characteristics makes it an ideal material for the development of photocatalytic membranes [141]. The photocatalytic performance using this material can be greatly enhanced by the formation of a heterojunction between graphene/GO and g-C_3_N_4_. This combination effectively improves the light absorption and enhances the separation of charges, leading to improved overall efficiency [142]. Moreover, the amalgamation of g-C_3_N_4_ and rGO not only addresses the issue of the grain boundary effect but also enhances the absorption of visible light. Additionally, it promotes the smooth flow of electrons and the separation of charges across the composite interface. Furthermore, the inclusion of rGO has proven advantageous in enhancing the flexibility and mechanical robustness of the membrane [143]. In a similar vein, the rGO membrane with g-C_3_N_4_ insertion demonstrated a notable enhancement in water permeability, all the while upholding a commendable photo-degradation capability [144]. Although a plethora of carbon-based photocatalytic membranes have been studied for the construction of photocatalytic membrane reactors, insignificant research has been carried out for the study of the feasibility of carbon NP-based photocatalytic MD. Moreover, it is important that the retentate obtained from a single MD process contains a high concentration of the contaminants that were rejected. As a result, further treatment is required for environmental purposes. The utilization of a photocatalytic membrane reactor combined with MD can effectively address this issue. In comparison to conventional pressure-driven methods, a photocatalytic membrane reactor coupled with MD offers the benefit of minimizing membrane fouling. However, it is important to note that complete mineralization of the organic pollutants may not be achievable. Instead, various intermediate compounds, both volatile and non-volatile, may be generated and pass through to the permeate, potentially exhibiting higher toxicity levels than the original contaminants. Therefore, it is essential to monitor the toxicity of the wastewater, treated water, and permeate to ensure environmental safety [140].

## 8. Challenges and Outlook

The field of nanotechnology has made significant progress in the development of various nanomaterials, particularly carbon-based nanomaterials and their derivatives. These nanomaterials possess exceptional properties that surpass those of conventional materials in the MD process. By integrating these nanoparticles into MD membranes, the shortcomings of traditional membrane materials are effectively addressed, bridging the gap between their limitations and the requirements of modern applications. Carbon nanomaterial-based membranes can effectively position themselves as cheaper alternatives to other NPs. The appealing properties offered by the carbon NPs have led to the development of the various novel carbon-based membranes in water treatment and MD. Carbon-based membranes in the MD process have demonstrated enhanced performance which includes increasing the flux, and improving the membrane’s mechanical, antifouling, and antibacterial properties. Despite the numerous benefits they offer, scaling them up for real-world applications remains a difficult challenge. The technological advancement of MD lags behind that of other established systems. Therefore, to be used in commercial applications, additional efforts are needed to enhance the chemical compatibility and physical stability of carbon-based membranes. Membranes reinforced with carbon nanomaterials suffer from poor dispersion and distribution, as well as weak interfacial interactions between the different surfaces of the carbon nanomaterials and the matrix. Like other nanoparticles, the non-uniform dispersal of carbon nanomaterials can lead to pore blocking in the membrane, hindering the progress of MD. It is crucial to develop an effective membrane synthesis method that meets the requirements for successful membrane modification. Moreover, certain types of carbon-based membranes are delicate and present considerable challenges in terms of handling and application. A key obstacle in their commercialization lies in ensuring the stability of NPs across the active membrane layer to prevent their leaching into the downstream environment. The potential risks posed by carbon nanomaterials to human health and the environment raise important concerns that need to be addressed. It is essential to develop strategies for monitoring the leaching of carbon-based materials, considering factors such as the leaching time and rate. One critical aspect of utilizing the MD process for large-scale operations involves integrating MD with other processes and utilizing a more affordable energy source to enhance its cost-effectiveness, environmental friendliness, and efficiency. The operational expenses associated with the MD process could be significantly reduced by utilizing alternative, cost-effective heat sources like waste heat and solar energy. From an energy perspective, the use of renewable solar energy presents an appealing option for the water industry. The progress in utilizing carbon nanomaterials for photothermal and photocatalytic MD has the potential to open new avenues for innovative processes that leverage both heat transfer and self-cleaning properties. Nevertheless, a thorough investigation is required to bridge the existing gap between the theoretical concept and practical applications, facilitating the swift transition of technology from the academic setting to industrial settings. Collaborative efforts across different disciplines, involving experts from the membrane field and materials science, will play a pivotal role in the realization of efficient next-generation carbon-based membranes aimed at addressing the global challenge of water scarcity. The photothermal efficiency of carbon nanomaterial layers on the membrane surface is influenced by its thickness and concentration. Increasing the thickness of the NP layers can enhance the light-to-heat conversion efficiency. However, this thicker photothermal layer may negatively impact the permeating fluxes by blocking the membrane pores and increasing the resistance to water-vapor mass transfer. Therefore, further research is required to develop photothermal materials that possess effective light absorption and efficient light-to-heat conversion properties. While high photothermal efficiency can be achieved in laboratory-scale setups, the thermal efficiency of pilot-scale setups utilizing natural light still requires improvement [117]. Simulation and real-time monitoring are essential to obtain insights into the fundamental behavior and kinetics of the light absorption, vapor generation, transport, and thermal diffusion of the photocatalytic MD process. It is of the utmost importance to discover cost-effective membrane materials and manufacturing methods to create membranes with excellent hydrophobicity and a superior thermal and chemical stability for applications in MD. Utilizing 3D printing in conjunction with various surface engineering techniques has the potential to enhance the performance of this process by fabricating distinctive structures that reduce mass transfer resistance and prevent fouling. Enhancements in the reproducibility and uniformity of the printed membranes are necessary to uphold the consistency of a satisfactory MD performance.

## 9. Conclusions

The primary objective of this review was to examine the various carbon-based nanomaterials that are presently employed in the advancement of cutting-edge MD membranes. Through a thorough analysis of the existing literature, it has been determined that the incorporation of carbon nanoparticles significantly enhances the hydrophobic properties of the composite membrane, leading to a decrease in fouling and wetting tendencies. The utilization of carbon-based nanomaterials has opened novel possibilities for MD membrane technology. The unique characteristics and simple synthesis procedure of carbon NPs, especially GO, rGO, and CNTs, further encourage their use in multifunctional applications in membranes, like in photocatalytic MD, photothermal MD, electro-responsive MD, etc. Compared to pristine membranes, carbon composite-based MD membranes present significant benefits through the augmentation of the membrane surface roughness. The advantages of carbon-based MD, in contrast to the use of other nanoparticles, include an increase in flux, a reduction in fouling, and the enhancement of mechanical properties and heat recovery. Carbon nanoparticles exhibit a synergistic improvement in both structural and functional characteristics when utilized as filler materials. However, it is important to acknowledge certain limitations associated with their use, such as scalability challenges and potential toxicity concerns.

## Figures and Tables

**Table 1:** Composition of polymer and carbon nanomaterials, methods of integration, application, and performance evaluation of some of the state-of -the-art MD processes mentioned in the literature.

Polymer Type	Carbon Nanomaterial	Incorporation Method	Application	Performance	References
--	BP (self-supporting)		DCMD	12 LMH/99% rejection	[52]
PVDF	GO-ODA		AGMD	16.7LMH/98.3% rejection	[58]
PVDF-HFP	CNTs	Electrospinning	DCMD	48.1 LMH/99.98% rejection efficiency	[64]
PVDF	APTS functionalized GO	Blending	AGMD	6.2 LMH	[96]
PTFE	CNTs	Coating	Electric enhance DCMD	7.89 LMH	[106]
PVDF-Co-HFP	CNTs	Electrospraying	DCMD	8.1 LMH	[107]
PVDF	GO/PVA&PP	Vacuum filtration	DCMD	52 LMH	[108]
PVDF	GO	Vacuum Filtration	AGMD	10.7 LMH	[109]
PVDF-CO-HFP	rGO	Electrospinning	DCMD	27.79LMH/100% rejection	[110]
PTFE	GO/PVDF	Drop cast	DCMD	97 LMH	[111]
PVDF-HFP	Powdered activated carbon	Chemical vapor deposition	DCMD	77 LMH/99% rejection	[112]
